# Real-World Cardiotoxicity in Metastatic Melanoma Patients Treated with Encorafenib and Binimetinib

**DOI:** 10.3390/cancers16172945

**Published:** 2024-08-23

**Authors:** Sidsel Pedersen, Marc Østergaard Nielsen, Marco Donia, Inge Marie Svane, Bo Zerahn, Eva Ellebaek

**Affiliations:** 1 Center for Cancer Immune Therapy, Department of Oncology, Copenhagen University Hospital, Herlev and Gentofte, 2730 Herlev, Denmark; marco.donia@regionh.dk (M.D.); inge.marie.svane@regionh.dk (I.M.S.); eva.ellebaek.steensgaard@regionh.dk (E.E.); 2 Department of Clinical Physiology and Nuclear Medicine, Copenhagen University Hospital, Herlev and Gentofte, 2730 Herlev, Denmark; marc.oestergaard.nielsen@regionh.dk (M.Ø.N.); bo.zerahn@regionh.dk (B.Z.)

**Keywords:** metastatic melanoma, BRAF and MEK inhibitors, cardiotoxicity, real-world data

## Abstract

**Simple Summary:**

Targeted therapies with BRAF- and MEK-inhibitors have significantly improved outcomes for patients with metastatic melanoma, but they come with a risk of heart-related side effects. This study included 108 real-world patients with metastatic melanoma from Eastern Denmark from 2019–2022 treated with encorafenib and binimetinib. Heart function was monitored with MUGA scans at baseline and every three months. While 18% of the patients experienced minor heart issues without symptoms, only 6% faced major problems, some needing medical intervention. However, no severe heart issues occurred beyond six months of starting the treatment. This suggests that it might be safe to reduce heart monitoring after six to nine months if no issues appear early on.

**Abstract:**

Modern therapies targeting the BRAF gene mutation in advanced melanoma have significantly improved patient outcomes but pose cardiovascular risks. This retrospective study in Eastern Denmark (2019–2022) assessed 108 melanoma patients treated with encorafenib and binimetinib. Patients were monitored for heart function using multigated acquisition (MUGA) scans. The study defined major cardiotoxicity as a decline in left ventricular ejection fraction (LVEF) by more than 10 percentage points to below 50%, and minor cardiotoxicity as a decrease in LVEF by more than 15 points but remaining above 50%. Results showed that 19 patients (18%) developed minor cardiotoxicity and were asymptomatic, while 7 (6%) experienced major cardiotoxicity, with two requiring intervention. Notably, no significant declines in LVEF were observed after six months of treatment. The study concluded that significant cardiotoxicity occurred in 6% of cases, mostly asymptomatic and reversible, and suggests that monitoring LVEF could potentially be reduced after 6–9 months if no early signs of cardiotoxicity are detected. This provides valuable insights into the cardiac safety of these treatments in real-world settings.

## 1. Introduction

Modern systemic therapies, including immunotherapy and targeted therapy, have significantly improved outcomes for patients with advanced melanoma [1,2,3]. Approximately 50% of melanomas harbor a mutation in the BRAF gene, which leads to the constitutive activation of the MEK-ERK signaling pathway, resulting in increased cell proliferation and growth [4]. Most BRAF mutations are point mutations substituting valine at amino acid 600 (V600), which can be targeted with selective BRAF inhibitors (BRAFi) [5] such as vemurafenib, dabrafenib, or encorafenib. BRAFi are used in combination with MEK inhibitors (MEKi); cobimetinib, trametinib, or binimetinib, as multiple studies have demonstrated improved progression-free survival (PFS) and overall survival (OS) with combination therapy compared to BRAFi monotherapy [1,6,7,8].

However, treatment with BRAFi/MEKi is associated with a risk of cardiovascular adverse events, including hypertension, prolongation of the QTc-interval, and a decrease in left ventricular ejection fraction (LVEF) [9,10,11,12]. Clinical studies have reported a decline in LVEF in approximately 8% of patients treated with BRAFi/MEKi [6,7,8,13]. The pathophysiological mechanism by which BRAFi/MEKi affect LVEF is not fully understood; it is assumed to be mostly associated with MEKi, given that the rate of cardiotoxicity is higher with combination therapy than with BRAFi alone [9]. Evidence suggests that activation of the MEK-ERK signaling pathway is critical for cardiomyocyte homeostasis and the cardiac stress response [14,15,16], and thus, inhibition of this pathway in healthy cardiomyocytes may cause cardiac dysfunction.

Although rare, a decrease in LVEF is considered a severe and potentially life-threatening adverse event, necessitating monitoring of cardiac function during treatment with BRAFi/MEKi. In a retrospective analysis from our center, we found a rate of cardiotoxicity induced by treatment with dabrafenib and trametinib comparable to that reported in clinical studies, with no clinically significant decreases in LVEF observed after the six-month evaluation [17]. The objective of this study was to examine the incidence and characteristics of cardiotoxicity induced by encorafenib and binimetinib as well as to confirm prior findings from the study of dabrafenib and trametinib.

## 2. Methods

### 2.1. Study Population

This study included real-world patients with advanced melanoma who were treated with encorafenib and binimetinib in the Eastern part of Denmark, covering approximately 45% of all Danish patients, from 1 May 2019 to 1 May 2022. Patients with advanced melanoma in the Eastern part of Denmark receive treatment at Herlev and Gentofte Hospital, thus representing an unselected regional cohort of Danish patients with metastatic melanoma. Patients were identified using the Danish Metastatic Melanoma Database (DAMMED), which comprises comprehensive data on all patients undergoing systemic treatment for advanced melanoma in Denmark [18].

Inclusion criteria encompassed stage III–IV unresectable melanoma, treatment with the BRAFi/MEKi combination of encorafenib and binimetinib, and a multigated acquisition (MUGA) scan for assessment of LVEF at baseline (performed max. 30 days before and 14 days after the first day of treatment with BRAFi/MEKi), as well as at least one MUGA scan for monitoring cardiac function approximately three months after initiation of therapy. Patients lacking a baseline MUGA scan, or with only one MUGA scan in total, were excluded from the analyses. Additionally, patients who switched from encorafenib and binimetinib to dabrafenib and trametinib before the first evaluation MUGA scan due to non-cardiac toxicities (e.g., skin or gastrointestinal toxicity) were also excluded.

In a previous analysis of cardiotoxicity during BRAFi/MEKi treatment, multiple potential risk factors for development of a decline in LVEF were considered, but no significant associations were found [17]. Consequently, an analysis of risk factors was not included in this study.

### 2.2. Assessment of Cardiac Function

Cardiac function was monitored using MUGA scans at baseline and subsequently every three months during therapy. MUGA scans were conducted as equilibrium radionuclide angiographies (ERNA) on a dedicated cardiac Cadmium Zinc Telluride (CZT) SPECT camera using ^99m^Tc-labelled human serum albumin (^99m^Tc-HSA) as a tracer. The methodology is described in detail in a previous publication [17]. The advantages of this method compared to echocardiography include low interobserver variation (coefficient of variance 1.7%) [19] and high practicability (98.2%) [20,21]. The recorded variables for each scan included LVEF, left ventricular end-systolic volume (LVESV), left ventricular end-diastolic volumes (LVEDV), left ventricular peak emptying rate (LVPER) and left ventricular peak filling rate (LVPFR). Additionally, data on height, weight, heart rate, and blood pressure were documented.

### 2.3. Cardiotoxicity

Major cardiotoxicity was defined as a minimum of a 10 percentage point (pp) decline in LVEF to <50%, while minor cardiotoxicity was defined as a reduction in LVEF of ≥15 pp but remaining >50% [22]. Patients with an LVEF <50% at baseline were analyzed separately. Cases with cardiotoxicity were further analyzed according to reversibility. Full recovery was defined as an LVEF increase to the baseline value, partial recovery as an LVEF reaching within 10 pp of the baseline level, and no reversibility as an LVEF remaining >10 pp below baseline.

### 2.4. Statistical Analysis

Continuous variables are reported as means ± standard deviation or medians according to normal distribution, and categorical variables expressed as frequencies and percentages.

OS was defined as the time between the date of first treatment with encorafenib and binimetinib to the date of death or last follow-up. Kaplan–Meier estimation was performed using the log-rank method, and plots were generated using GraphPad Prism version 5. A *p*-value < 0.05 was considered statistically significant.

## 3. Results

### 3.1. Study Population

A total of 108 patients with advanced melanoma treated with encorafenib and binimetinib were included in this study. Of these, 61% were male, and the mean age was 63.5 years. Additionally, 83% of the patients had a performance status of 0–1, and the mean LVEF at baseline was 70%. The baseline characteristics are summarized in Table 1.

### 3.2. Cardiotoxicity

**Figure 1:**
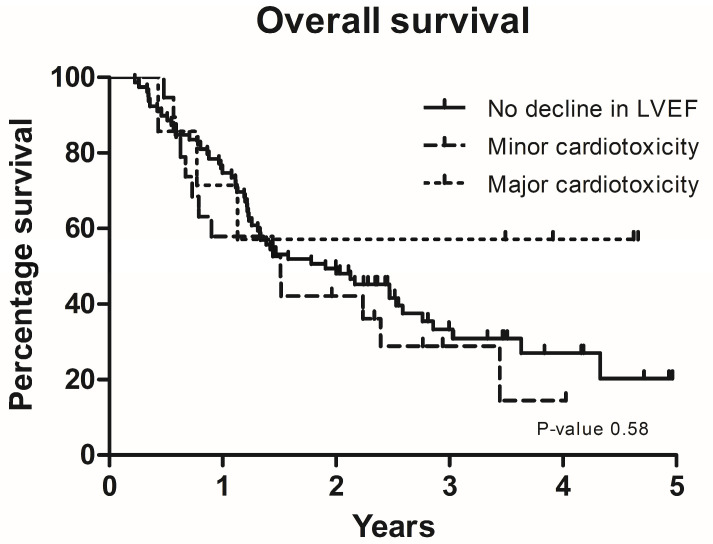
Overall survival in years for patient with no decline in left ventricular ejection fraction (LVEF), minor cardiotoxicity and major cardiotoxicity.

In total, 26 patients experienced cardiotoxicity; 19 patients (18%) experienced minor cardiotoxicity, while 7 patients (6%) experienced major cardiotoxicity. Three patients presented with an LVEF below 50% at baseline. There was no significant difference in OS among patients with minor cardiotoxicity, major cardiotoxicity, or no cardiotoxicity (Figure 1).

### 3.3. Minor Cardiotoxicity

Of the 19 patients with minor cardiotoxicity, two had a dose reduction due to the decrease in LVEF in accordance with national treatment guidelines. All patients were asymptomatic, and none required consultation with a cardiologist.

### 3.4. Major Cardiotoxicity

Among the seven patients with major cardiotoxicity, two had cardiac symptoms, while five were asymptomatic. One patient with symptoms of heart failure was hospitalized and diagnosed with non-ST-segment elevation myocardial infarction (NSTEMI) and aortic stenosis; this patient underwent successfully surgery for both conditions and achieved a full recovery of LVEF. Encorafenib and binimetinib were discontinued; however, due to cancer progression, the patient was later reintroduced to BRAFi/MEKi therapy with dabrafenib and trametinib with no subsequent impact on LVEF. The other patient with cardiac symptoms had a dose reduction and was evaluated by a cardiologist who found no signs of heart failure; the patient continued encorafenib and binimetinib at a reduced dose with no further decrease in LVEF or alterations in treatment.

Of the five asymptomatic patients with major cardiotoxicity, two continued treatment without alterations, whereas treatment was temporarily paused for the remaining three patients. Two of these three patients were evaluated by a cardiologist, who found no signs of cardiac disease. All three resumed encorafenib and binimetinib in reduced doses without further decreases in LVEF.

Among the seven patients with major cardiotoxicity, four achieved full recovery of LVEF, and three achieved partial recovery.

### 3.5. LVEF <50% at Baseline

The three patients with a LVEF <50% at baseline all received encorafenib and binimetinib in a reduced dose (66% of the full dose) and maintained this dosage without any decline in LVEF.

### 3.6. Time to Cardiotoxicity

The median time from initiation of treatment with encorafenib and binimetinib to the diagnosis of a decrease in LVEF for patients developing any cardiotoxicity was 90 days and 134 days for patients with major cardiotoxicity only (Table 2), corresponding to the time of the first evaluation scan. The decline in LVEF was detected at the first or second evaluation scan (within 6 months of treatment) for six out of the seven patients with major cardiotoxicity. One patient experienced the decline in LVEF later than six months; this patient had no cardiovascular symptoms but was evaluated by a cardiologist, who found no signs of cardiac disease and concluded that the patient had a stable low LVEF. The patient continued treatment with encorafenib and binimetinib without any alterations and experienced no symptoms of cardiotoxicity. For 16 out of the 19 patients (84%) with minor cardiotoxicity, the decrease in LVEF was detected at the first or second evaluation scan (within the first 6 months of treatment). Three patients experienced a decrease in LVEF later than six months; none of them had cardiac symptoms or required treatment alterations due to the decline in LVEF.

### 3.7. Changes in Paraclinical Values

A decline in LVEF, defined as cardiotoxic, was characterized by an increase in LVESV and accompanied by a decrease in LVPFR and LVPER, regardless of whether the cardiotoxicity was minor or major. Heart rate did not change significantly during treatment. Changes in paraclinical values between baseline and the lowest LVEF (nadir) during treatment with encorafenib and binimetinib are presented in Table 3.

## 4. Discussion

In this retrospective study of real-world patients with advanced melanoma, we found that 24% of patients treated with encorafenib and binimetinib experienced a decline in LVEF during treatment, with 6% experiencing a clinically significant decline to an LVEF <50%.

A decline in LVEF during treatment with encorafenib and binimetinib was very common. Eighteen percent of patients experienced minor cardiotoxicity; all were asymptomatic, and none required consultation with a cardiologist. For 90% of these patients, the decline in LVEF did not necessitate any alterations in treatment, while two patients had a dose reduction; however, whether this was necessary remains unknown. More than half of the patients with minor cardiotoxicity had a baseline LVEF of >80%, which is above the normal level [23]. Therefore, a decline of 15 pp is not necessarily pathological nor indicative of heart failure, suggesting that a decline in LVEF to a value >50% might not be clinically significant given the method used, since LVEF measured with CZT-ERNA has a higher upper limit of normal compared to echocardiography (ECHO). Considering only a decline in LVEF to a value <50% clinically significant, the rate of cardiotoxicity for real-world patients with advanced melanoma receiving encorafenib and binimetinib was 6%, which aligns with the rates reported in both clinical trials [6,8,13] and other retrospective cohorts [12,24]. Compared to patients eligible for clinical trials, real-world patients often have, e.g., more comorbidities, greater age, and higher PS, and therefore are possibly more fragile and more susceptible to adverse events; however, the rates of cardiotoxicity in this real-world study were similar to those reported in clinical trials, indicating that patients not eligible for clinical trials do not have a higher risk of cardiotoxicity.

Two of the patients with major cardiotoxicity had clinical symptoms, and both experienced full recovery of LVEF following relevant treatment. In one case, the cardiac symptoms and the decline in LVEF were most likely due to a preexisting cardiac condition rather than solely cardiotoxicity induced by encorafenib and binimetinib, as the patient was diagnosed with NSTEMI and aortic stenosis and underwent surgery for both, resulting in full recovery of the LVEF. However, we cannot exclude treatment with encorafenib and binimetinib as a contributing cause. The other patient with major cardiotoxicity, who experienced cardiac symptoms with mild dyspnea and palpitations, was evaluated by a cardiologist who found no signs of heart failure; the symptoms subsided spontaneously, and LVEF recovered fully.

Three patients had a baseline LVEF below 50%. These patients received encorafenib and binimetinib at a reduced dose and did not experience any further decline in LVEF during therapy. While it is not possible to conclusively determine if patients with preexisting cardiac dysfunction could receive the full dose of encorafenib and binimetinib, these results suggest that with careful monitoring of cardiac function and symptoms, a baseline LVEF <50% should not be an absolute contraindication for treatment with BRAFi/MEKi.

Importantly, no patients experienced a clinically significant decline in LVEF beyond six months after initiating treatment with encorafenib and binimetinib. One patient experienced major cardiotoxicity more than six months after initiation of treatment. However, the decrease in LVEF was asymptomatic and, upon cardiological evaluation, was interpreted as a stable low LVEF; the patient continued therapy without alterations, and the decline in LVEF was not considered clinically relevant. Our analysis of cardiotoxicity during treatment with dabrafenib and trametinib [17] also found no clinically significant declines in LVEF beyond six months after initiation of treatment. Based on these findings, we suggest that monitoring of LVEF could be halted 6–9 months after initiation of therapy with BRAFi/MEKi, provided the patient has no cardiac symptoms and has not experienced a decline in LVEF within the first six months of treatment.

ECHOs are often used to monitor heart function during cardiotoxic treatment. The results from this study using MUGA scans for monitoring heart function are most likely applicable in monitoring of heart function with ECHOs as well, as the high sensitivity that the MUGA provides might not be necessary in order to identify a significant decline in LVEF. However, this needs to be reaffirmed in future studies.

In a previous analysis of real-world patients with metastatic melanoma receiving dabrafenib and trametinib, 11% of patients experienced a decline in LVEF to a value <50% [17]. This rate of cardiotoxicity is slightly higher compared to the rate of cardiotoxicity found in the current study; however, due to the small sample size, statistical significance cannot be demonstrated. The main cardiac variable driving the decline in LVEF was an increase in LVESV, which was seen along with decreasing LVPER and LVPFR. This was also observed in cardiotoxicity induced by dabrafenib and trametinib, indicating no apparent difference in pathophysiological mechanisms causing cardiotoxicity during treatment with either drug combination. In the analysis of cardiotoxicity during treatment with dabrafenib and trametinib, we found no association between cardiotoxicity and specific baseline characteristics, such as comorbidity, ECG abnormalities, body mass index, etc. Due to these previous negative findings and the small subgroups of patients in this study tainting a potential analysis of predictive factors with a high degree of uncertainty, an analysis on predictive factors was not conducted.

A major limitation of this study is the limited number of patients, especially when analyzing subgroups of patients with cardiotoxicity. Moreover, comparing two historically different cohorts is inherently challenging. Nonetheless, CZT-ERNA provides improved reproducibility compared to standard methods for monitoring cardiac function, potentially mitigating the negative effects of a limited sample size for both cohorts.

## 5. Conclusions

In conclusion, 6% of real-world patients with advanced melanoma treated with encorafenib and binimetinib experienced a decline in LVEF of more than 10 pp to a value <50%. All cases of cardiotoxicity showed full or partial reversibility, and all clinically significant declines in LVEF occurred within the first six months of treatment.

## Figures and Tables

**Table 1:** Baseline characteristics.

Baseline Characteristics	All Patients	Patients with No LVEF Decline	Patients with Minor Cardiotoxicity	Patients with Major Cardiotoxicity	Patients with LVEF <50% at Baseline
Number of patients	108	79	19	7	3
Sex, male	66 (61)	52 (66)	9 (47)	4 (57)	1 (33)
Age (mean ± SD)	63.4 ± 13.7	62.2 ± 13.4	63.9 ± 15.8	68.6 ± 13.1	53.6 ± 4.5
BMI (mean ± SD)	24.8 ± 4.1	24.9 ± 4.1	25.4 ± 4.8	22.7 ± 1.9	23.9 ± 3.2
Performance status:					
0–1	90 (83)	67 (85)	17 (90)	4 (57)	2
≥2	18 (17)	12 (15)	2 (11)	3 (43)	1
Brain metastases	42 (39)	31 (39)	5 (26)	4 (57)	2
LDH					
<ULN	53 (50)	40 (51)	8 (44)	4 (57)	1
>ULN	53 (50)	38 (49)	10 (56)	3 (43)	2
Missing	2	1	1	0	–
BRAF/MEKi as 1. line treatment	59 (55)	43 (54)	10 (53)	4 (57)	2
ICI in later lines	31 (53)	24 (56)	5 (50)	0	2 (100)
BRAF/MEKi as 2. line or higher	49 (45)	36 (46)	9 (47)	3 (43)	1 (33)
ICI in previous lines	49 (100)	36 (100)	9 (100)	3 (100)	1 (100)
ICI in later lines	20 (41)	16 (44)	3 (33)	1 (33)	–
Comorbidities					
Hypertension	23 (21)	18 (23)	4 (21)	1 (14)	–
Ischemic heart disease	4 (4)	1 (1)	–	3 (43)	–
Diabetes	3 (3)	2 (3)	–	1 (14)	–
COLD	10 (9)	5 (6)	3 (16)	2 (29)	–
MUGA values (means ± SD)					
LVEF (%)	70 ± 12	69 ± 10	82 ± 7	58 ± 6	40 ± 10
LVEDV (mL)	86 ± 24	88 ± 24	73 ± 16	90 ± 31	106 ± 28
LVESV (mL)	28 ± 16	29 ± 14	14 ± 6	39 ± 18	65 ± 27
HR (beats per minute)	73 ± 14	72 ± 14	78 ± 14	75 ± 19	68 ± 7
Systolic BP (mmHg)	126 ± 17	126 ± 18	125 ± 14	124 ± 16	130 ± 20
Diastolic BP (mmHg)	75 ± 10	76 ± 9	71 ± 11	75 ± 12	80 ± 11
LVPER (mL/s)	−3.5 ± 1.1	−3.4 ± 1.0	−4.4 ± 0.9	−2.6 ± 0.5	−2.2 ± 0.8
LVPFR (mL/s)	2.9 ± 1.0	2.8 ± 0.8	3.8 ± 1.4	2.6 ± 0.7	2.0 ± 0.6
LVPER adjusted (mL)	−4.7 ± 1.6	−4.7 ± 1.7	−5.7 ± 1.1	−3.5 ± 0.6	−3.3 ± 1.3
LVPFR adjusted (mL)	4.0 ± 0.9	3.9 ± 0.8	4.7 ± 1.0	3.5 ± 0.5	3.0 ± 1.0

Abbreviations: BMI, body mass index; BP, blood pressure; BRAF/MEKi, BRAF/MEK inhibitors; COLD, chronic obstructive lung disease; HR, heart rate; ICI, immune checkpoint inhibitor; LDH, lactate dehydrogenase; LVEF, left ventricular ejection fraction; LVEDV, left ventricular end diastolic volume; LVESV, left ventricular end systolic volume; LVPER, left ventricular peak emptying rate; LVPFR, left ventricular peak filling rate; MUGA, multigated acquisition; SD, standard deviation; ULN, upper limit of normal.

**Table 2:** Time to cardiotoxicity from the date of first treatment to the date of the MUGA scan with a decline in left ventricular ejection fraction (LVEF).

Patient Group	Mean Time to Decline in LVEF (Days)	Median Time to Decline in LVEF (Days)	Range (Days)
All patients with cardiotoxicity	174	90	71–1246
Minor cardiotoxicity	179	78	71–1246
Major cardiotoxicity	160	134	76–377

**Table 3:** Change in paraclinical values between the MUGA scan performed at baseline and the evaluation MUGA with the lowest left ventricular ejection fraction (LVEF) performed during treatment with encorafenib and binimetinib.

Patient Group	LVEF (%)	LVEDV (mL)	LVESV (mL)	LVPER (mL/s)	LVPFR (mL/s)	LVPER-Adj (mL)	LVPFR-Adj (mL)	HR s-1
No LVEF decline (n = 76)	−6 ± 4.8 (−8%)	6 ± 15.3 (10%)	7 ± 6.7 (42%)	1 ± 0.9 (−13%)	0 ± 0.6 (−11%)	0 ± 0.9 (−8%)	0 ± 0.7 (−7%)	−4 ± 10.9 (−4%)
Minor cardiotoxicity (n = 22)	−19 ± 3.3 (−24%)	7 ± 16.6 (12%)	16 ± 6.9 (158%)	1 ± 1 (−29%)	−1 ± 1 (−28%)	1 ± 1.1 (−24%)	−1 ± 1 (−22%)	−6 ± 13.2 (−6%)
Major cardiotoxicity (n = 7)	−14 ± 3.8 (−24%)	11 ± 15.6 (13%)	17 ± 6.3 (49%)	1 ± 0.5 (−22%)	−1 ± 0.6 (−24%)	1 ± 0.7 (−17%)	−1 ± 0.6 (−22%)	−6 ± 15.5 (−4%)

Abbreviations: Adj, adjusted; HR, heart rate; LVEF, left ventricular ejection fraction; LVEDV, left ventricular end diastolic volume; LVESV, left ventricular end systolic volume; LVPER, left ventricular peak emptying rate; LVPFR, left ventricular peak filling rate; MUGA, multigated acquisition.

## Data Availability

The data that support the findings of this study can be made available via application to the Danish Metastatic Melanoma Database steering committee. Further details are available from the corresponding author upon request.
